# Intracardiac bone cement embolism resulting in ventricular perforation: an unusual cause of sudden chest pain

**DOI:** 10.1093/icvts/ivac292

**Published:** 2023-01-09

**Authors:** Martin O Schmiady, Mathias Possner, Thomas Horisberger, Ahmed Ouda

**Affiliations:** Clinic for Cardiac Surgery, University Heart Center, University Hospital Zurich, Zurich, Switzerland; Department of Cardiology, Kantonsspital St Gallen, St Gallen, Switzerland; Institute of Anaesthesiology, University Hospital Zurich, Zurich, Switzerland; Clinic for Cardiac Surgery, University Heart Center, University Hospital Zurich, Zurich, Switzerland

**Keywords:** Bone cement, Polymethylmethacrylate, Ventricle perforation, Percutaneous kyphoplasty

## Abstract

Leakage of bone cement is a known complication after percutaneous kyphoplasty. In rare cases, bone cement can reach the venous system and cause life-threatening embolism. We present the case of a 73-year-old male, who was admitted to our hospital with new-onset chest pain and dyspnoea. He had a history of percutaneous kyphoplasty. Multimodal imaging showed intracardiac cement embolism in the right ventricle with penetration of the interventricular septum and perforation of the apex. The bone cement was successfully removed during open cardiac surgery.

## INTRODUCTION

Reinforcement of vertebral fractures with polymethylmethacrylate (PMMA) bone cement through percutaneous kyphoplasty is a therapeutic procedure, to obtain pain relief and mechanical stability of the vertebral body. Although uncommon, bone cement can leak into the vertebral venous plexus, which is connected to the azygos venous system, reaching the vena cava, right heart chambers and pulmonary arterial system. In this report, we present a case of intracardiac cement embolization, resulting in symptomatic perforation through the interventricular septum, 2 years after percutaneous kyphoplasty.

## CASE REPORT

**Figure 1:**
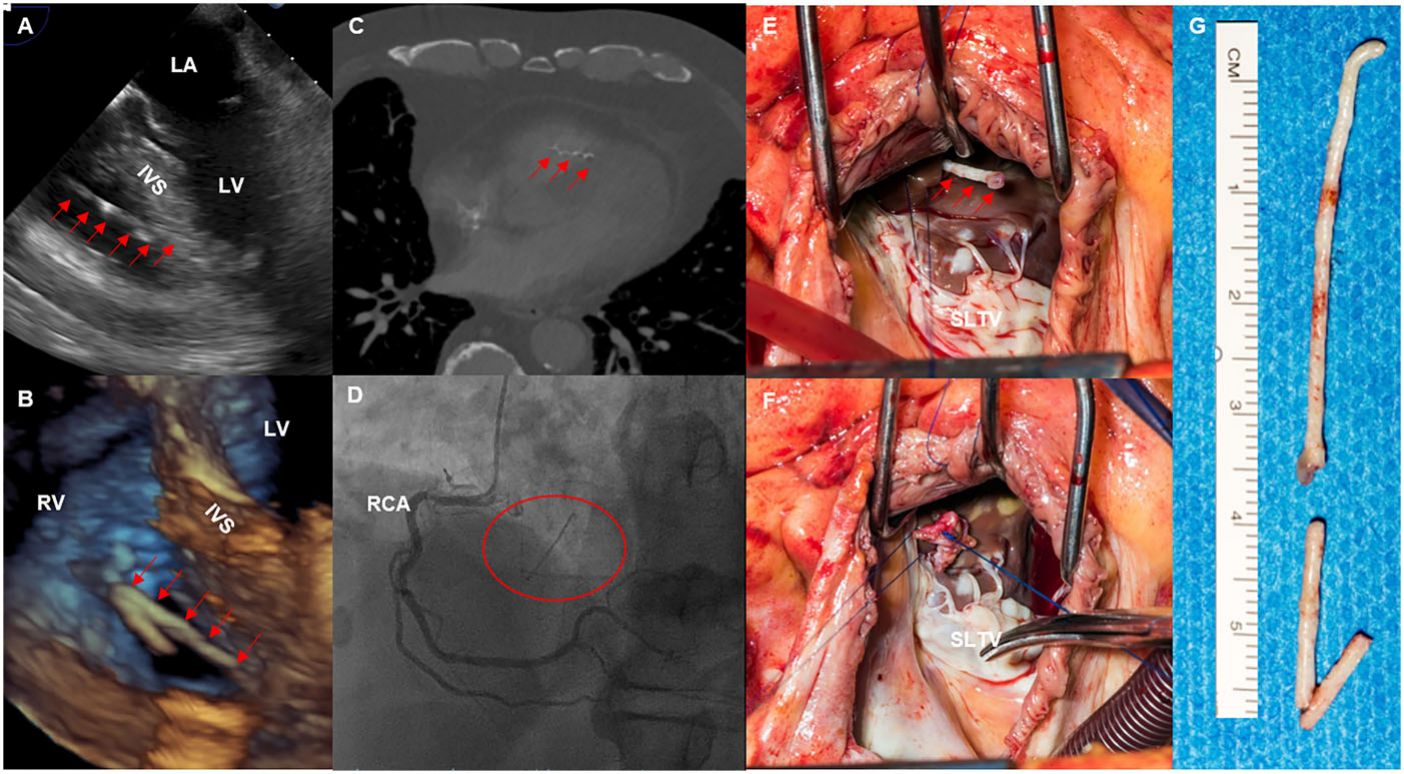
(**A** and **B**) Perioperative transoesophageal echocardiography showing the foreign body in the right ventricle, penetrating the interventricular septum (arrows). (**C**) Computed tomography scan showed a 6.5-cm-long cement fragment in the right ventricle. (**D**) Coronary angiogram, revealing the cement fragments in the right ventricle (circle). (**E** and **F**) Surgical view through the right atrium on the foreign body (arrows). After removal, the entry into the interventricular septum was closed, using a pericardial patch (septal leaflet of the tricuspid valve). (**G**) Bone cement after surgical removal.

A 73-year-old male presented with sudden onset chest pain and dyspnoea to our emergency department. He had a history of percutaneous kyphoplasty for traumatic L2 vertebral fracture 2 years ago. Beside an elevated blood pressure (145/80 mmHG) and sinus tachycardia (100 beats/min), the physical examination was normal. Blood tests showed normal levels of cardiac enzymes. Electrocardiogram exhibited normal sinus tachycardia without ST-segment deviation. A chest computed tomography was performed and ruled out aortic dissection and pulmonary embolism. Surprisingly a needle-like foreign body was seen in the right ventricle, penetrating the interventricular septum and perforating the apex into the pericardial space (Fig. 1C). Echocardiography confirmed the findings of a 6.5-cm-long structure in the right ventricle (Fig. 1A and B). Due to symptomatic perforation, decision was made for surgical removal. Preoperative coronary angiography showed a 70% stenosis of the left main, which had to be addressed during surgery. During fluoroscopy, the foreign material could be visualized in the right ventricle (Fig. 1D). Operation was performed through a median sternotomy using moderate hypothermic (34°C) cardiopulmonary bypass and single-shot 100 ml Cardioplexol^®^ (Swiss Cardio Technologies, Bichsel, Interlaken) cardioplegic solution into the aortic root. The right atrium was opened, releasing the view to a rod-shaped, white foreign body, penetrating the ventricular septum, below the insertion of the septal tricuspid valve leaflet (Fig. 1E). The foreign material was removed and the entry point sewn over with a pericardial patch (Fig. 1F and G). The left anterior descending and circumflex artery were bypassed using arterial grafts. Postoperative course was uneventful and the patient was discharged 10 days after surgery.

## DISCUSSION

Bone cement containing PMMA is frequently used among various orthopaedic procedures. PMMA is an inert material, which is not reabsorbed in the human body. Cement leakage is reported to be as high as 65% in the treatment of vertebral fractures [1]. It may cause local complications (cord or nerve root compression) or systemic complications such as pulmonary, intracardiac and paradox embolism [2]. Although often occurring immediately after the orthopaedic procedure, cement embolism can present as a late complication many years after the initial intervention [3]. In case of symptoms or intracardiac localization, removal of the foreign material is recommended, because of the potential risk of perforation and sudden death due to tamponade or severe valve dysfunction. In high-risk patient, endovascular treatment can be discussed, bearing the risk of incomplete removal and pulmonary embolism after accidental fragmentation [4].

## CONCLUSION

The present case highlights that bone cement embolism can occur even years after kyphoplasty. Precise cardiac work-up, using multimodality imaging, is essential for diagnosis and surgical planning. Because of the risk of potentially life-threatening complications, surgical removal is indicated in the case of intracardiac embolism.

## COMPLIANCE WITH ETHICAL STANDARDS

All procedures performed in this case were in accordance with the ethical standards of the institutional and/or national research committee and with the 1964 Helsinki Declaration and its later amendments or comparable ethical standards. Informed consent was obtained from the patient included in this work.

## Data Availability

I confirm that all data about the described case are vailable and can be delivered after request.
